# Supplementary material for: Dermoscopy of Small Diameter Melanomas with the Diagnostic Feasibility of Selected Algorithms—A Clinical Retrospective Multicenter Study
Source: Cancers (Basel). 2021 Dec 3;13(23):6095. doi: 10.3390/cancers13236095 (PMC8656839; doi:10.3390/cancers13236095)
Supplement: Supplementary file 1 [file cancers-13-06095-s001.zip › cancers-1447385-supplementary.pdf]

Supplementary Materials: Dermoscopy of Small Diameter Melanomas with the Diagnostic Feasibility of Selected Algorithms—A Clinical Retrospective Multicenter Study

Monika Slowinska, Grazyna Kaminska-Winciorek, Elzbieta Kowalska-Oledzka, Iwona Czarnecka, Robert Czarnecki, Anna Nasierowska-Guttmejer, Elwira Paluchowska and Witold Owczarek

Table S1. Review of the literature regarding published studies which included microM cases. Presented data summarize the methodology, results and conclusions of the studies addressing dermoscopic diagnostic of microM [2,3,5–20].

Study [Ref.]	microM cases	Diameter cut off [mm]	TNM Invasiveness	Study group	Limitation	Purpose of study/Results / Conclusions
Bono et al. 1999[1]	47	≤6 mm (2–6 mm; median 5)	pTis *n* = 14; pT1 *n* = 33;	microM; no comparator	Single center<br>No comparator<br>Retrospective analysis<br>No statistical analysis of dermoscopic structures of microM preformed	Purpose of study: frequency of occurrence of microM in clinical context.<br>17% microM of 270 cases of consecutive melanoma 50% (23 lesions) of microM were clinically diagnosed (ABCD rule, positive when fulfilled more than 50% of criteria).36 microM were evaluated dermoscopicaly and diagnosed in 72% (26 lesions) (positive when 1 criterium was found of: radial streaming, pseudopods, grey-blue veil, whitish veil, black dots et the periphery if network present, thick irregular network, regression,<br>erythema, milky red background with red dots).<br>Combination of clinical and dermoscopic examination revealed 86% microM.
Pizzichetta et al. 2001 [2]	8	≤5 mm	pTis *n* = 37; ≥pT1 *n* = 53;	M in situ (*n* = 37) (size <5mm *n* = 8; > 5–10 mm *n* = 20; > 10 mm (*n* = 9) vs. Invasive M (*n* = 53)	Multicenter study<br>Retrospective analysis<br>No statistical analysis of dermoscopic structures of microM vs.. >5 mm melanoma preformed<br>Small sample size of microM	Puropose of study: dermoscopic structures of melanomas in situ vs. invasive.<br>Dermoscopic structures chose for evaluation: pigment network alterations, irregular extensions and branched streaks, gray-blue areas, extensions pseudopods, brown globules, black dots, blue-whitish veil, hypopigmentation, white scar-like areas, and linear and dotted vascular patterns.<br>Frequency of features in MpTis: blue-white veil (78%), gray-blue areas (76%), black dots (73%), and irregular extensions and branched streaks (62%) white scar-like areas (0), linear and/or dotted vascular patterns (0).
Carli et al. 2003 [3]	10	< 6 mm	pTis *n* = 24; ≥ pT1 *n* = 40; (median 0.3 mm)	Melanocytic lesions *n* = 200 divided into groups:<br>- small <6 mm (nevi *n* = 48, M *n* = 10),<br>- intermediate 6-9mm (nevi *n* = 61, M *n* = 26), -	Single center<br>Retrospective analysis<br>Small sample size of microM<br>No statistical analysis of dermoscopic structures of microM	Purpose of the study: analysis of the melanoma diameter influence on its dermoscopic detection.<br>Diagnostic sensitivity in group <6mm 60% (SD 25.6) vs. 63.7% (SD 28.2) for clinical vs. clinical and dermoscopy.<br>Specificity in group < 6mm 85.4% (SD 8.0) vs. 82.0% (SD 10.0)) for clinical vs. clinical and dermoscopy.

				large ≥10 mm (nevi n = 28, M n = 27)		Diagnostic accuracy in group < 6mm 80.6% (SD 6.4) and 78.8% (SD 10.1)) for clinical vs. clinical and dermoscopy.
Bono et al. 2004 [4]	22	≤3 mm		microM; no comparator	Single center No comparator Retrospective analysis No statistical analysis of dermoscopic structures of microM preformed	Purpose of the study: to assess the clinical and dermoscopic features of microM of 3 mm. MicroM are dark, black macule, even color, with well-defined borders; asymmetric or symmetric in shape. Sensitivity of clinical examination 50%; dermoscopic -100%.
Fernandez et al. 2004 [5]	94	≤ 6 mm	Median thickness: 0.29 mm in small M group; 0.4 mm in > 6 mm	>6mm M	Single center Retrospective analysis of pathological reports No statistical analysis of dermoscopic structures of microM preformed	Purpose of the study: frequency of microM based on analysis of pathological reports. 38.21% melanomas ≤ 6 mm microM average age 52.45 years vs. >6mm M 59.16 years ($p < 0.002$), microM less invasive (0.29–0.4 mm, $p < 0.02$).
Helsing et al. 2004 [6]	18	<7 mm (3–6 mm; mean 4.6)	pTis n = 47; pT1 n = 56; >pT1 n = 55; median thickness in small M 0.8mm	>7mm n = 140	The Norwegian Melanoma Project 1990-1993 Retrospective analysis of the medical registry No statistical analysis of dermoscopic structures of microM preformed	Purpose of the study: assess the frequency and prognosis of melanomas <7 mm. The frequency of microM 11.4%. Diagnostic sensitivity for microM 44%.
Bono et al. 2006 [7]	23	≤3 mm (1–3 mm; median 2)	pTis n = 4; pT1 n = 19;	microM; no comparator	Single center Prospective study No comparator No statistical analysis of dermoscopic structures of microM preformed	Purpose of the study: to analyze the sensitivity and specificity of clinical and dermoscopic examinations microM ≤3 mm. Clinical diagnosis - based on the subjective experience of the single clinician vs. dermoscopic criteria for melanoma – Menzies' method. Sensitivity: dermoscopy (Menzies' method) vs. clinical examination 83% vs. 43% ($p < 0.01$). Specificity 69% Menzies' method vs. 91% clinical examination ($p < 0.001$).
Friedman et al. 2008 [8]	49	≤ 6 mm	pTis n = 28; ≥pT1 n = 21;	Pigmented skin lesions; n = 941 Cases from the commercial digital dermoscopic database	Blinded comparison study No statistical analysis of dermoscopic structures of microM preformed	Purpose of the study: to assess the diagnostic sensitivity of dermoscopists vs. an automatic multispectral computer-vision system in diagnosing pigmented skin lesions <6 mm. The prevalence of small MM 25%. microM average sensitivity 40% (median 43%); average specificity 80% (median 84%). MicroM pTis sensitivity 33%, microM invasive 48% (thickness 0.1–1.4mm; median 0.32mm).
Abbasi et al. 2008 [9]	35	≤ 6 mm (2–6 mm)	pTis n = 84 (1.5% <6 mm);	Pigmented lesions </> 6 mm Total n = 1657;	Cohort study Prospective analysis	Purpose of the study: to assess the influence of the M diameter </> 6mm on clinical diagnosis based on ABCD rule.

			≥pT1 *n* = 54 (2.6% <6 mm);	*n* = 804 <6mm	No statistical analysis of dermoscopic structures of microM preformed	Frequency of microM<6mm 4.1%. Diagnostic sensitivity: 5 mm – 83%; 6 mm - 75%; 7 mm - 63%) - decreases with increase of the diameter of lesions. Diagnostic specificity: 5 mm – 38%, 6 mm – 54%, 7 mm - 65%) - increases with the diameter. The study supported importance of D – criterion >6mm of ABCDE rule in early M diagnosis.
De Giorgi et al. 2012 [10]	34	<6 mm (3–6mm)	pTis *n* = 18; pT1 *n* = 15 >pT1 *n* = 1;	microM; Melanocytic nevi *n* = 69	Single center Retrospective analysis	Purpose of the study: to analyze the clinical and dermatoscopic features of small (<6mm) pigmented melanocytic lesions. Clinical criteria – ABCD rule vs. dermoscopic pattern analysis. Dermoscopic structures: atypical pigment network, irregular dots, irregular streaks, irregular globules, irregular diffuse pigmentation, regression structures, atypical vascular pattern, bule-white vel- all frequency differences were $p < 0.05$ with exclusion of irregular dots. Dermoscopic pattern: reticular 17.7% vs. 33.3%, globular 2.9% vs. 14.5%, starburst 2.9% vs. 4.4%, homogenous 0 vs. 9%, unspecific 76.5% vs. 39.1% ($p < 0.001$). The clinical criteria for diagnosing melanoma are not as reliable in the diagnosis of pigmented lesions of less than 6 mm diameter in comparison to the dermoscopy.
Pupelli et al. 2013 [11]	24	≤5 mm (2–5 mm)	pTis *n* = 11 pT1 *n* = 10 >pT1 *n* = 3	Melanocytic nevi ≤5 mm *n* = 72	Two center Prospective analysis	Purpose of study: analysis of morphological differences in reflectance confocal microscopy (RCM) structures between the melanoma and melanocytic naevi ≤5 mm. Dermoscopic criteria of the 7-point checklist vs. RCM criteria Combination of dermoscopic and confocal structures reduced the false positive diagnoses of melanoma. Dermoscopic structures of microM vs. nevi with p<0.05: atypical vessels, irregular pigmentation, irregular dots/globules, peripheral streaks, regression. The 7-point checklist score ≥3 in 22/24 (92%) microM, no statistically significant difference to nevi (46%)
Seidenari et al. 2014 [12]	22<br><br>57	≤4 mm (2.4–4 mm; mean 3.4)<br><br>4–6 mm	pTis n = 135 (*n* = 10 for microM); >pT1 n = 347 (*n* = 12 for microM);	MM>4 mm; *n* = 460	Two center Retrospective analysis	Purpose of the study: comparison of the dermoscopic features of MMs <4 mm and >4mm Dermoscopic patterns of 22 microM ≤4 mm: multicomponent 32%, bicomponent 27%, spitzoid 18%, reticular 14%, globular and island 5%. Dermoscopic features of 22 microM ≤4 mm: atypical network 77%, irregular

						dots/globules 55%, irregular streaks 36%, regression 32%, irregular pigmentation 32%, atypical vessels and blue-white veil 5%, asymmetry 36%.<br>Score of 7-point mean 3.5 (SD 1.41) in ≤4 mm microM and mean 3.84 (SD 1.65) in 4–6 mm melanomas were significantly lower than in larger melanomas.<br>The statistically significant differences between <6mm vs. >6mm melanomas were found for: asymmetry, number of colors, atypical vessels, irregular globules/dots and regression.
Emiroglu et al. 2014 [13]	29	< 6 mm	pTis n = 13;<br>pT1 n = 58;<br>> pT1 n = 13	M > 6 mm n = 42;<br>location restricted to trunk;	Multicenter<br>Retrospective analysis<br>Location of melanoma restricted to trunk	Purpose of the study: frequency of dermatoscopic features in trunk melanomas </> 6mm pTis vs. invasive.<br>Dermoscopic melanoma-specific features in microM: asymmetry (79.3%), blotches (79.3%), variety of colors (72.4%), atypical dots and globules (72.4%) atypical pigment network (69.0%), blue-grey veil (65.5%, $p < 0.05$), multicomponent structure (58.6%, $p < 0.05$), streaks (58.6%), regression structures (48.3%, $p < 0.05$), milky red areas (24.1%, $p < 0.05$), atypical vessels (13.8%).<br>No correlations between dermoscopic features and melanoma invasiveness were found.
Salerni et al. 2015 [14]	8	<5 mm (2.5–4.5 mm; mean of 3.7).	pTis n = 6;<br>pT1 n = 2;	M restricted to lower limbs;<br>No comparator	Single center<br>Retrospective analysis<br>Small sample size of microM<br>No comparator<br>Location of melanoma on lower limbs<br>Type: Case series	Purpose of the study: dermoscopic features of microM on lower limbs.Dermoscopy evaluation by the pattern analysis:7/8 reticular pattern and atypical network; 5/8 asymmetry; 3/8 radial streaks/ pseudopods;3/8 only 1color, 2/8 2 colors, 3/8 > 2 colors among microM.
Dika et al. 2017 [15]	62	<6mm<br>n = 31<br>86.11% of DDM<br><br>n = 31<br>58.49% of EM<br>(p = 0.01)	Total number of patients<br>pTis n = 22 (75% DDM);<br>pT1 n = 67 ();	Difficult to diagnose M (DDM) </>6mm vs. evident M (EM) </> 6mm;	Single center<br>Retrospective analysis<br>Location of melanoma on lower limbs<br>Lack of results of dermoscopic analysis regarding microM <6mm in DDM and EM	Purpose of the study: dermoscopic differentiation between melanoma simulating melanocytic nevi (difficult-to-diagnose; DDM) and evident melanomas (EM).<br>DDM features: depigmentation" as only specific criterion, risk factor correlated with DDM – M pTis (adj. OR 12.61; 95 % CI 1.92–82.65) and M <0.4 mm thickness (adj. OR 8.58; 95 % CI 1.27–58.00).<br>The melanoma diameter (>6 mm vs. <6 mm) was statistically not significant in the multivariate analysis.<br>Dermoscopic features differences DDM vs. EM with p<0.05: multicomponent pattern 0% vs. 15%, depigmentation 47.2% vs. 15%, scar-like depigmentation 0% vs. 18.9%, multiple color 0% vs. 45.3%, veil 8.3% vs. 30.2%, lack of melanoma-specific criteria 43% vs. 3%.

						The diagnostic sensitivity of the dermoscopic algorithms in M below ≤1mm thickness:<br>- pattern analysis 64.05%,<br>- the 7-point checklist 61.08%,<br>- Menzies' method 57.30%,<br>- ABCD 42.70%.
Drugge et al. 2018 [16]	19<br>9<br>27	≤ 2mm<br>2 -≤ 3mm<br>>3 - ≤ 6mm	pTis *n* = 50;<br>≥ pT1 *n* = 31;	Melanocytic lesions *n* = 268;<br>M *n* = 81	Single center<br>Retrospective analysis<br>No statistical analysis of dermoscopic structures preformed<br>Type: Letter to editor	Purpose of the study: report of frequency of microM detected based on comparison of total body photography sets and dermoscopy in follow-up of patients.<br>Number needed to excise 3.1 M vs. 12.01 nevi.<br>Comparison of complete sets of TBP images with dermoscopy may reveal microM
Megaris et al. 2018 [17]	26	2–5mm; (mean 3.5mm)	pTis *n* = 19;<br>pT1 *n* = 7;	microM;<br>no comparator	Single center<br>Retrospective analysis<br>No comparator<br>Small sample size<br>Limited statistical analysis<br>Type: letter to editor	Purpose of the study: description of dermoscopic morphology of microM.<br>Dermoscopic pattern: globular 0%, reticular 57.7%, starburst 3.8%, structureless 26.9%, mixed (reticular and globular) 11.5%, multicomponent 0%.<br>Dermoscopic melanoma-specific structures: irregular dots/globules 88.4%, irregular hyperpigmented areas 88.4%, polygones / angulated lines 46.2%, atypical network 42.3%, irregular blotch 38.4%, negative network 11.5%, prominent skin markings 11.5%. regression 7.6%, shiny white structures 3.8%, blue-white veil 3.8%.
Campos-do-Carmo G et al. 2021 [28]	123	1–6 mm;<br>48.2% up to 3 mm<br>91.5% up to 5 mm	pTis *n* = 69;<br>pT1 *n* = 27;<br>atypical melanocytic proliferations/ incipient melanomas; *n* = 27	481 melanocytic lesion ≤ 6mm located on trunk and limb;<br>358 melanocytic nevi ≤ 6mm as a comparator	Two center<br>Prospective analysis<br>Location of melanoma restricted to trunk and limbs	Purpose of the study: evaluate clinical and dermatoscopic features in suspicious pigmented cutaneous lesions ≤6mm and to calculate their diagnostic feasibility.<br>The independent variables for microM <6mm, adjusted for age, gender and location: streaks (adjusted Odds Ratio [aOR] 2.5; 95% CI 1.3–4.7; $p = 0.006$), and structureless area (aOR 2.2, 95% CI 1.2–4.0, $p = 0.011$). The symmetric typical pigment network was a protection variable (aOR 0.4, 95% 0.7–0.9, p = 0.040).<br>The clinical diagnosis of microM (ABCDE rule) in 33.5% of the lesions (36.6% sensitivity, 67.6% specificity). The dermatoscopic diagnosis of microM in 90.0% of lesions (92.7% sensitivity, 11.2% specificity, 81.6% NPV). 29.3% of confirmed melanomas had both a clinical and dermatoscopic diagnosis of melanoma.<br>The modified ABC-point list algorithm (Blum, Rassner and Garbe ; 2003): score ≥4 in 54.3% of microM (sensitivity 61.8, specificity 48.3%, accuracy 51.8%).

Abbreviations and acronyms used in supplementary table:

microM	micromelanoma
M	melanoma
TNM	T (tumour), N (node), M (metastasis)
SD	standard deviation
DDM	difficult-to-diagnose melanoma
EM	evident melanoma
CI	confidence interval
TBP	total body photography
NPV	negative predicted value
OR	odds ratio
TDS	total dermoscopy score of the dermoscopic ABCD rule of Stolz
ABC	Modified ABC-point list of dermoscopy (algorithm) by Blum et al.
ABCD	the dermoscopic ABCD rule of Stolz (algorithm)
ABCDE	asymmetry (A), borders (B), colors (C), diameter >6 mm (D), evolution (E) – the clinical rule to diagnose melanoma by Friedman et al.
TADA	Triage Amalgamated Dermoscopic Algorithm by Rogers et al.
7-point	7-point checklist of dermoscopy by Argenziano et al.
Vs.	versus

References

1. Bono, A.; Bartoli, C.; Moglia, D.; Maurichi, A.; Camerini, T.; Grassi, G.; Tragni, G.; Cascinelli, N. Small melanomas: A clinical study on 270 consecutive cases of cutaneous melanoma. *Melanoma Res.* **1999**, *9*, 583–586.
2. Pizzichetta, M.A.; Argenziano, G.; Talamini, R.; Piccolo, D.; Gatti, A.; Trevisan, G.; Sasso, G.; Veronesi, A.; Carbone, A.; Soyer, H.P. Dermoscopic criteria for melanoma in situ are similar to those for early invasive melanoma. *Cancer* **2001**, *91*, 992–997. https://doi.org/10.1002/1097-0142(20010301)91:5<992::AID-CNCR1089>3.0.CO;2-I.
3. Carli, P.; De Giorgi, V.; Chiarugi, A.; Nardini, P.; Mannone, F.; Stante, M.; Quercioli, E.; Sestini, S.; Giannotti, B. Effect of lesion size on the diagnostic performance of dermoscopy in melanoma detection. *Dermatology* **2003**, *206*, 292–296. https://doi.org/10.1159/000069939.
4. Bono, A.; Bartoli, C.; Baldi, M.; Moglia, D.; Tomatis, S.; Tragni, G.; Cascinelli, N.; Santinami, M. Micro-melanoma detection. A clinical study on 22 cases of melanoma with a diameter equal to or less than 3 mm. *Tumori* **2004**, *90*, 128–131.
5. Fernandez, E.M.; Helm, K.F. The diameter of melanomas. *Dermatol. Surg.* **2004**, *30*, 1219–1222. https://doi.org/10.1111/j.1524-4725.2004.30379.x.
6. Helsing, P.; Loeb, M. Small Diameter Melanoma: A Follow-Up of the Norwegian Melanoma Project. *Br. J. Dermatol.* **2004**, *151*, 1081–1083. https://doi.org/10.1111/j.1365-2133.2004.06248.x.
7. Bono, A.; Tolomio, E.; Trincone, S.; Bartoli, C.; Tomatis, S.; Carbone, A.; Santinami, M. Micro-melanoma detection: A clinical study on 206 consecutive cases of pigmented skin lesions with a diameter < or = 3 mm. *Br. J. Dermatol.* **2006**, *155*, 570–573. https://doi.org/10.1111/j.1365-2133.2006.07396.x.
8. Friedman, R.J.; Gutkowicz-Krusin, D.; Farber, M.J. The Diagnostic Performance of Expert Dermoscopists vs. a Computer-Vision System on Small-Diameter Melanomas. *Arch. Dermatol.* **2008**, *144*, 476–482. https://doi.org/10.1001/archderm.144.4.476.
9. Abbasi, N.R.; Yancovitz, M.; Gutkowicz-Krusin, D.; Panageas, K.S.; Mihm, M.C.; Googe, P.; King, R.; Prieto, V.; Osman, I.; Friedman, R.J.; et al. Utility of lesion diameter in the clinical diagnosis of cutaneous melanoma. *Arch. Dermatol.* **2008**, *144*, 469–474. https://doi.org/10.1001/archderm.144.4.469.
10. de Giorgi, V.; Savarese, I.; Rossari, S.; Gori, A.; Grazzini, M.; Crocetti, E.; Sara Longo, A.; Oranges, T.; Massi, D. Features of small melanocytic lesions: Does small mean benign? A clinical-dermoscopic study. *Melanoma Res.* **2012**, *22*, 252–256. https://doi.org/10.1097/CMR.0b013e3283527430.
11. Pupelli, G.; Longo, C.; Veneziano, L.; Cesinaro, A.M.; Ferrara, G.; Piana, S.; Moscarella, E.; Ricci, C.; Zalaudek, I.; Seidenari, S.; et al. Small-diameter melanocytic lesions: Morphological analysis by means of in vivo confocal microscopy. *Br. J. Dermatol.* **2013**, *168*, 1027–1033. https://doi.org/10.1155/2014/206260.
12. Seidenari, S.; Ferrari, C.; Borsari, S.; Fabiano, A.; Bassoli, S.; Giusti, F.; Ponti, G.; Magnoni, C. Dermoscopy of small melanomas: Just miniaturized dermoscopy? *Br. J. Dermatol.* **2014**, *171*, 1006–1013. https://doi.org/10.1111/bjd.12542.
13. Emiroglu, N.; Pelin Cengiz, F.; Hofmann-Wellenhof, R. Dermoscopic and clinical features of trunk melanomas. *Postępy Dermatologii Alergologii* **2014**, *31*, 362–367. https://doi.org/10.5114/pdia.2014.47119.
14. Salerni, G.; Alonso, C.; Fernández-Bussy, R. A series of small-diameter melanomas on the legs: Dermoscopic clues for early recognition. *Dermatol. Pract. Concept.* **2015**, *5*, 8. https://doi.org/10.5826/dpc.0504a08.
15. Dika, E.; Chessa, M.A.; Ribero, S.; Fanti, P.A.; Gurioli, C.; Lambertini, M.; Baraldi, C.; Patrizi, A. Diagnostic Efficacy of Digital Dermoscopy and Clinical Findings in Thin Melanoma of the Lower Limbs. *Acta Derm. Venereol.* **2017**, *97*, 1100–1107. https://doi.org/10.2340/00015555-2705.

16. Drugge, E.D.; Volpicelli, E.R.; Sarac, R.M.; Strang, S.R.; Elston, D.M.; Drugge, R.J. Micromelanomas identified with time-lapse total body photography and dermoscopy. *J. Am. Acad. Dermatol.* **2018**, *78*, 182–183. https://doi.org/10.1016/j.jaad.2017.07.049.
17. Megaris, A.; Lallas, A.; Bagolini, L.P.; Balais, G.; Cuevas, R.G.; Lallas, K.; Gkentsidi, T.; Manoli, S.M.; Papageorgiou, C.; Spyridis, I. et al. Dermatoscopic features of melanomas with a diameter up to 5mm (micro-melanomas): A retrospective study. *J. Am. Acad. Dermatol.* **2020**, *83*, 1160–1161. https://doi.org/10.1016/j.jaad.2020.04.006.
18. Campos-do-Carmo, G.; Nobre, A.B.; Cuzzi, T.; Argenziano, G.; Ferreira, C.G.; Thuler, L.C.S. Melanocytic lesions ≤ 6mm: Prospective series of 481 melanocytic trunk and limb lesions in Brazil. *PLoS ONE* **2021**, *16*, e0252162. https://doi.org/10.1371/journal.pone.0252162.
